# Otakuism and the Appeal of Sex Robots

**DOI:** 10.3389/fpsyg.2019.00569

**Published:** 2019-03-29

**Authors:** Markus Appel, Caroline Marker, Martina Mara

**Affiliations:** ^1^Human-Computer-Media Institute, University of Würzburg, Würzburg, Germany; ^2^Johannes Kepler University Linz, LIT Robopsychology Lab, Linz, Austria

**Keywords:** sex robots, anime, manga, fan culture, otakuism, shyness, uncanny valley

## Abstract

Social robots are becoming increasingly prevalent in everyday life and sex robots are a sub-category of especially high public interest and controversy. Starting from the concept of the *otaku*, a term from Japanese youth culture that describes secluded persons with a high affinity for fictional manga characters, we examine individual differences behind sex robot appeal (*anime and manga fandom*, *interest in Japanese culture*, *preference for indoor activities*, *shyness*). In an online-experiment, 261 participants read one out of three randomly assigned descriptions of future technologies (*sex robot*, *nursing robot*, *genetically modified organism*) and reported on their overall evaluation, eeriness, and contact/purchase intentions. Higher *anime and manga fandom* was associated with higher appeal for all three future technologies. For our male subsample, sex robots and GMOs stood out as shyness yielded a particularly strong relationship to contact/purchase intentions for these new technologies.

## Introduction

For many years now, robots have been involved in the production of cars, chemicals, and other industrial goods. More recently, tech companies have started to develop robots that are meant to be applied outside factory walls, in hospitals, shopping malls, and in people’s homes. The vision of a proliferation of robotic technologies has been met with mixed reactions (Gnambs and Appel, 2019); human-like robots in particular have been the cause of skepticism and negative emotions among potential users. The arguably most dazzling category of these social robots—and one that captures a large portion of media attention (e.g., Kleeman, 2017; National Broadcasting Company [NBC] news, 2017; Awford, 2019)—is sex robots. The aim of this work is to examine individual differences as predictors of affective responses and behavioral intentions toward sex robots.

Our starting point to identify explicative individual difference dimensions is the cultural phenomenon of the *otaku* (e.g., Kinsella, 1998; Galbraith, 2009; Ito et al., 2012; Kam, 2013). Prototypical members of this group are male and socially secluded. They have a strong attachment to Japanese manga comics and sometimes even label fictional human-like manga characters as their waifus (their ‘wives’). The merchandise targeted at otakus include body pillows with manga character prints and interactive holographic animations of their waifus. As it happened with related concepts such as the “nerd” or the “geek” (cf. McCain et al., 2015), the term otaku has lost much of its stigmatization in recent times. Many self-ascribed otakus in- and outside of Japan nowadays proudly describe themselves as part of this community (Galbraith, 2009).

As outlined in the following sections, we examined a set of individual difference variables associated with the subculture of *otakus* (anime and manga fandom, interest in Japanese culture, preference for indoor activities, shyness). These variables were expected to be predictors of the eeriness elicited by sex robots, as well as respondents’ overall evaluations, and purchase intentions with respect to this technology. Using an experimental design, we disentangle sex robot appeal from responses to non-sexual communication robots and non-robotic innovations.

### Sex Robots

A sex robot can be described as a mechanical doll of human-like appearance and size that is able to perform intercourse by means of motor-equipped artificial genitalia (cf. Choi, 2008; Pearson, 2015; Richardson, 2016). Whereas a high degree of autonomy or artificial intelligence may not be mandatory, advanced prototypes of sex robots still are able to carry out simple verbal communication, simulate different personalities, and express enjoyment in order to increase their anthropomorphic realism (e.g., Abyss Creation’s *Harmony* and *Henry*). Historically, human sexuality has been a driver of communication technology acceptance and use (Barss, 2010), and sexuality is a field of application that could accelerate the proliferation of emerging technologies, such as virtual reality (VR) or social robotics.

Unsurprisingly, the matter of sex robots is a controversial one. Some scholars have expressed worries about ethical issues and spoken out for restrictions on their further development. They fear a potential reinforcement of objectification, gender stereotypes, and non-empathetic forms of sexual encounter to come along with the rise of sex robots (Sullins, 2012; Richardson, 2016). In contrast, others see potential psychological and social benefits of sexual robot partners, for instance in sexual therapy or as a less critical alternative to human sex workers (Levy, 2007, 2008; Devlin, 2015). Market prognoses are divided about if and when sex robots will be available for a broader public. Some forecasts estimate, however, that we will start to see them in high-income households by 2025 already and that human–robot sex will overtake interpersonal sex by 2050 (Levy, 2008; Pearson, 2015). According to Levy (2008) and Cheok et al. (2016), even love relationships between persons and robotic dolls might become a reality by then. Nevertheless, it remains yet largely unaddressed if society in general and the targeted user groups in particular are actually accepting of sex robots. The robots’ visual design arguably constitutes an influential factor here. Making sex puppets as lifelike as possible may result in user responses that follow Mori’s uncanny valley hypothesis. According to this framework, highly realistic yet not perfectly human-like robots likely elicit an experience of discomfort and eeriness in human observers (Mori, 1970; Mori et al., 2012; cf. Mara and Appel, 2015; Lischetzke et al., 2017).

Aside from robot-specific factors, individual differences on the users’ side likely play a key role in the appeal of sex robots. Evidence on the role of user variables is rare, however. To the best of our knowledge, only two studies have addressed user responses to sex robots empirically to date. In one exploratory study, men were more accepting of sex robots than women in many potential areas of usage (e.g., for sex education, instead of cheating, for unusual sex practices). Men were more open toward unusual robot appearances (e.g., celebrity, fantasy figure, animal), and more interested in a personal try-out than women (Scheutz and Arnold, 2016). There is little evidence, however, regarding user variables beyond gender, such as personality traits or interests as predictors of feelings, evaluations, and behavioral intentions toward sex robots. In a recent study (Szczuka and Krämer, 2017) only the attitude toward robots in general terms consistently predicted the sexual attractiveness of sex robots (more negative attitudes were associated with lower perceived attractiveness).

By connecting prior work from marketing, cultural studies, personality psychology, and work on the link between science-fiction and robot acceptance, we find *otakuism* to be a particularly intriguing starting point for identifying individual characteristics likely to be associated with the appeal of sex robots.

### Otakuism

Otakuism is a subcultural phenomenon that has started in Japan and that in recent years has become prevalent in the West (there are overlaps to the concept of geek or weeaboo). Otakus are characterized by their strong interest in the fictional worlds found in animation, manga, and games (AMG), along with a substantial affinity for new and future technologies – both fictional and non-fictional (Washida, 2005). *Otaku* 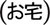 is the Japanese word for home or the house of another person. Otakus prefer to spend a remarkably large part of their time at home. Moreover, otakus are described as shy and less skilled with social contacts (Saitō, 2007; Kam, 2013). Finally, otakus in the West tend to be interested in Japan and Japanese culture. We conceive these aspects – AMG fandom, preference for indoor activities, shyness, and interest in Japanese culture – as dimensional rather than categorical, with meaningful variations for both genders and individuals of different age (Kinsella, 1998; Saitō, 2007). The dimensional approach follows prior work and operationalizations of otakuism in the marketing field (Niu et al., 2012).

It is important to emphasize that we do not distinguish between otakus and non-otakus as a binary decision. Rather, otakuism is represented by scores on the four continuous dimensions of AMG fandom, preference for indoor activities, shyness, and interest in Japanese culture. These scores can vary independently. Although we expect positive associations between the variables, an individual could score, for example, high on AMG fandom, and high for preference for indoor activities, but low on shyness and interest in Japanese culture. Moreover, our approach does not require individuals to self-ascribe being an otaku. Individuals scoring high the dimensions are not required to have a positive self-definition of being an otaku. They may even be unaware of the term itself as for some people the dimensions might be summarized in terms of *geek*, *nerd*, *anime and manga*, *Japanophile*, or *Weeaboo* culture.

### Otakuism Dimensions as Predictors of Sex Robot Appeal

We assume that the dimensions of otakuism (in concert with gender) could be predictors of the (lack of) appeal of sex robots, with appeal specified into eeriness, overall evaluation, and contact/purchase intention.

#### AMG Fandom

AMG fandom describes individual differences concerning the interest in and attachment to animation, comics, and games. For a prototypical otaku, these media involve Japanese manga and anime (Kinsella, 1998). Fans enjoy watching, reading or playing them, and they spend a substantial amount of money on these media products and related merchandise (Niu et al., 2012). Fans are familiar with the storylines and may join others in private or in public to express their attachment to the fictional AMG characters. Findings from culture studies suggest that some AMG fans develop a close (pseudo-)romantic affinity to one or more fictional characters which are referred to as the *waifu* or the *husbando* of the fan (Zeng, 2018). Humanoid robots with human feelings and behaviors are common in manga and anime. For example, a famous manga and anime is *Chobits*, which is about a love story between a humanoid robot and its owner. As previous literature showed, science fiction featuring humanoid robots can lead to higher willingness for buying and using real-life robots among recipients (Mara and Appel, 2015; Appel et al., 2016). AMG fans are supposed to have a higher familiarity with the concept of (pseudo-)romantic relationships with non-human characters (e.g., the waifu) as well as with storylines including robotic partners. Appel et al. (2016) suggest that technological innovations at times violate expectations as they lack a meaningful connection to previously formed mental representations of the world, oneself, or oneself in the world (cf. Heine et al., 2006). Following this line of thought, AMG fandom should be positively associated with sex robot appeal. More specifically, the stronger individuals’ AMG fandom, the less uncanny sex robots should be perceived (Hypothesis 1a). Moreover, a positive relationship between AMG fandom and overall evaluations of sex robots (Hypothesis 1b) and purchase intentions (Hypothesis 1c) was expected.

#### Interest in Japanese Culture

For otakus in Western societies, a penchant for Japanese culture is a constituting aspect of their subculture. Manga and anime originate from Japan as do many innovations in robotic technologies. Interest in Japanese culture goes beyond AMGs and includes other domains such as technology, food, or religion. Although it may not be far-fetched to connect shintoism or Japanese culture more generally to the acceptance of human-like robots (Shaw-Garlock, 2009), we addressed the link between the interest in Japanese culture and the appeal of sex robots as a research question. (Research Question 1).

#### Preference for Indoor Activities

Whereas some individuals like to go out and socialize at malls, in restaurants, pubs, or discotheques, or enjoy a long walk in the woods, go mushroom hunting or fishing, others prefer to stay at home. Otakus tend to habitually avoid outdoor activities. They rather engage in computer-mediated entertainment, write and draw for themselves or communicate through the Internet. Staying indoors makes it difficult for individuals to connect to others, including the initiation of romantic interactions. Moreover, having a relationship with a partner who prefers spending times outdoors could be aversive. A sex robot, who only functions indoors, could therefore be an attractive partner. Thus, we assume that a preference for indoor activities is associated with heightened sex robot appeal (Hypotheses 2a, 2b, and 2c for eeriness, overall evaluation, and purchase intentions, respectively).

#### Shyness

Shyness is characterized by discomfort and inhibition in the presence of other people (Jones et al., 1986). Shy individuals want to connect to others, but they believe they lack the skills and behavioral patterns to do so successfully. The initial stages of human interactions are experienced as highly uncomfortable, thus, shy people often avoid social situations such as dating (Arkowitz et al., 1978; Heimberg et al., 1980). Shyness is associated with more difficulties achieving face-to-face intimacy (Jones and Carpenter, 1986; Asendorpf, 2000). Shy individuals seem to feel more comfortable with computer-mediated contact provided by social networking sites and similar platforms, as the relationship between Facebook use and friendship quality increases with users’ shyness (Baker and Oswald, 2010). Based on these results, we assume that the use of sex robots could be particularly attractive for shy individuals. Self doubts and social anxious responses expected or actually experienced in intimate situations should be alleviated in human–robot interaction. Thus, we expected a negative association between shyness and the eeriness of sex robots (Hypothesis 3a) and positive associations between shyness and overall evaluations (Hypothesis 3b), and purchase intentions (Hypothesis 3c).

## Materials and Methods

Our aim was to design a study that allowed us to examine responses to sex robots specifically, rather than responses to humanoid robots or technological innovations more generally. To this end, an experimental design was used. We presented a description of a sex robot or a description of a different technological innovation, that is, a nursing robot or a genetically modified organism (GMO). We chose a nursing robot as a point of comparison, because this is a social robot domain that includes close personal contact to users, but lacks the sexuality aspect. We chose a GMO as a point of comparison, because this technology has no connection to sexuality, but it shares the hybrid character of sex robots and social robots more generally (human and machine). GMOs and sex robots are both hybrids that appear to violate essentialist theories of natural kinds (Wagner et al., 2010).

Statistically, our experimental approach allowed us to compare the strength of three relationships (or simple slopes): (a) associations between the focal individual difference dimensions and responses to sex robots, (b) associations between the same dimensions and a humanoid robot of a different domain, and (c) associations between the focal individual difference dimensions and responses to a non-robotic, but similarly hybrid technology.

### Participants

In our experiment members of the MTurk participant pool (restricted to United States residents) participated online. Research that investigated the quality of responses gained from Amazon’s MTurk yielded positive results (e.g., Buhrmester et al., 2011; Casler et al., 2013; Hauser and Schwarz, 2016). Data gathered from an MTurk sample was described as high quality, valid data (e.g., as compared to participant pool data, Hauser and Schwarz, 2016), providing a higher degree of diversity, and even allowing a valid operationalization of behavioral tasks (Casler et al., 2013). We cannot rule out that – as compared to the general population – MTurk workers might have somewhat higher scores on the otakuism dimensions (e.g., prefer being indoors).

On the MTurk platform potential participants could read the study title “Technology and Psychology” and the brief description “Academic survey. Answer questions about yourself and new technology.” For those who chose to participate, informed written consent was obtained from all participants. Of the 304 participants who completed the study, we excluded 30 participants who failed to respond correctly to a control question, asking for the technology described to them at an earlier part of the survey (see below). Another 13 participants worked for less than 100 s on the survey, indicating that they did not work thoroughly on the survey. The final sample consisted of *N* = 261 participants (107 female) with an average age of *M* = 34.66 years (*SD* = 10.42). Regarding power, we based our analysis on G^∗^Power linear multiple regression, fixed model R^2^ increase estimates (Faul et al., 2009), with a mid-sized effect (*f*^2^ = 0.15), given α = 0.05, power = 0.80, number of tested predictors = three interactions (between the categorical variables and one continuous dimension), and total number of predictors = 9. This analysis yielded a total required sample size of 78. Although the complexity of the design is not fully represented in this analysis we deemed the number of participants more than three times the identified sample size to be sufficient.

### Stimulus Material

The participants were randomly assigned to read one out of three short descriptions of a new technology. In the target condition, a human-like robot *Ellix* is introduced, with the main function of providing sexual pleasure (sex robot). In the first control condition, the human-like robot called *Ellix* is described as built for nursing (nursing robot). In the second control condition, a non-robotic innovation is described, genetically modified food (GMO, see Appendix for the complete descriptions).

### Measures

#### Otakuism

The four otakuism dimensions were measured with the help of a self-report questionnaire. A penchant for anime, manga, and games (AMG) was measured with three items adapted from Niu et al. (2012; short name of the dimension: *AMG fandom*). The item wordings were “I like watching anime (Japanese animation video),” “I often purchase products like manga, anime or games,” and “I like reading manga (Japanese comics).” The internal consistency for the scale was α = 0.86. The items on AMG fandom as well as the items pertaining to the three other otakuism dimensions went with a five-point scale (1 = *very uncharacteristic or untrue, strongly disagree*; 5 = *very characteristic or true, strongly agree*).

The second factor was preference for Japanese culture, e.g., its history or food (*Japanese culture*). The following items were used “I am interested in Japan’s history (e.g., Samurai, Geishas, Shinto religion and its myths),” “I am interested in Japanese culture,” “I am interested in Japanese food culture,” and “I am interested in Japanese society.” Scale reliability was good (α = 0.91).

The third factor was *preference for indoor activities*, measured with four items adapted from Niu et al. (2012). The items were “I prefer to spend my time with indoor activities like reading, watching movies, playing games,” “I like outdoor activities like meeting people, go hiking or shopping” (reversed), “I think I could fulfill all my leisure needs with my computer,” and “I don’t like to be in a big crowd.” Scale reliability was satisfactory (α = 0.71).

The fourth factor, *shyness*, was measured with the help of a well-established, 10-item shyness scale by Hopko et al. (2005), item example “It is hard for me to act natural, when I am meeting new people.” The reliability was good, as indicated by α = 0.92.

### Dependent Variables

First, we measured the *eeriness* the participants felt while reading about the robots (or the genetically modified organism). We asked them to rate their feelings on three items ‘uneasy,’ ‘unnerved,’ and ‘creeped out’ (Gray and Wegner, 2012). The items went with a five-point scale (1 = *not at all*; 5 = *extremely*). Internal consistency for this short scale was very good (α = 0.95). Second, the participants gave an *overall evaluation* of the new technology they read about. A semantic differential with a six-point scale ranging from -3 to +3 was used. The item pairs were ‘hate it – love it’; ‘negative – positive’; ‘repulsive – attractive’ (α = 0.98). *Behavioral intentions* were measured as contact and buying intentions. The two items were “I would avoid any contact with it” (reversed), “I can imagine that I would buy it if I had enough money.” Both items went with a five-point scale (1 = *not at all*; 5 = *extremely*), α = 0.79.

## Results

### Otakuism: Mean Differences Between Men and Women and Zero-Order Correlations

We first compared the mean scores for men and women (see Table 1, left columns). All four otakuism dimensions were not significantly affected by an individual’s gender (all *t*s < 1.14, *p*s > 0.25). With respect to correlations, anime fandom and Japanese culture as well as indoor activities and shyness were significantly related among both genders (see Table 1, right columns). Only for men, anime fandom and indoor activities, and anime fandom and shyness were substantially related, indicating that the otakuism network of related traits and interests might be tighter for men than for women.

**Table 1:** Otakuism dimensions: means, standard deviations, and correlations for both genders.

				Zero-order correlations			
		*M* (*SD*) Male	*M* (*SD*) Female	1	2	3	4
1	Anime fandom	2.22 (1.17)	2.07 (1.14)	–	0.58***	0.12	0.17
2	Japanese culture	3.20 (1.07)	3.10 (1.19)	0.47***	–	–0.05	0.08
3	Indoor activities	3.10 (0.97)	2.97 (0.87)	0.42***	0.26**	–	0.27**
4	Shyness	2.92 (0.95)	2.84 (0.91)	0.29***	0.10	0.45***	–


*^∗∗∗^*p* < 0.001; ^∗∗^*p* < 0.01. Correlations above the diagonal = results for the female subsample (*n* = 107). Correlations below the diagonal = results for the male subsample (*n* = 154).*

### Regression Analyses Outline

We conducted a series of hierarchical regression analyses to examine the role of the four otakuism dimensions on the appeal of sex robots and the alternative technological innovations of nursing robots and GMOs. The technology was dummy-coded (two coding variables with sex robots as the comparison group), so was gender (men = 0, women = 1). The gender and technology variables were entered first in the regression analysis (Step 1), followed by interactions between gender and technology variables (Step 2). Next, the focal otakuism dimension was entered (Steps 3a–3d). In a subsequent step, all two-way interactions between the otakuism dimension and technology and gender were included (Steps 4a–4d), and the two third-order interactions were entered last in the equation (Steps 5a–5d). Given the four otakuism dimensions, our main analyses comprised four regression equations for each of the three dependent variables. Results of additional analyses with otakuism dimensions as predictors entered together are displayed in the online (Supplementary Table S1).

### Effects of Technology and Gender

We first present the main effects of the technology treatment and gender for each of the three DVs (see Table 2, *Technology and Gender*). The sex robot was considered eerier than the nursing robot (*M* = 3.19, *SD* = 1.31; *M* = 2.22, *SD* = 1.25; *B* = -0.91, *SEB* = 0.19, *p* < 0.001), but it did not differ significantly from the GMO (*M* = 3.00, *SD* = 1.33; *B* = -0.10, *SEB* = 0.20, *p* = 0.60). Women reported stronger feelings of eeriness than men overall (*M* = 3.14, *SD* = 1.45; *M* = 2.56, *SD* = 1.25, *B* = 0.54, *SEB* = 0.16, *p* = 0.001).

**Table 2:** Regression analyses with otakuism dimensions as predictors and eeriness, overall evaluation, and purchase intentions regarding the innovation as criteria.

	Eeriness				Evaluation				Purchase intentions			
	*B*	*SEB*	β	*p*	*B*	*SEB*	β	*p*	*B*	*SEB*	β	*p*
**Technology/gender**												
*Step 1*												
Gender	0.54	0.16	0.19	0.001	–0.89	0.21	–0.24	<0.001	–0.41	0.15	–0.16	0.006
DY1 sex robot vs. nursing robot	–0.91	0.19	–0.32	<0.001	1.29	0.25	0.34	<0.001	0.86	0.18	0.33	<0.001
DY2 sex robot vs. GMO	–0.10	0.20	–0.04	0.599	–0.01	0.25	–0.00	0.981	0.30	0.18	0.11	0.098
*Step 2*												
DY1^∗^gender	–0.42	0.39	–0.10	0.288	0.46	0.50	0.09	0.352	0.45	0.35	0.12	0.209
DY2^∗^gender	–0.32	0.40	–0.08	0.419	1.03	0.51	0.18	0.044	0.49	0.36	0.13	0.176
**AMG fandom**												
*Step 3a*												
AMG fandom	–0.24	0.08	–0.18	0.002	0.10	0.17	0.16	0.003	0.28	0.07	0.22	<0.001
*Step 4a*												
DY1^∗^AMG fandom	–0.18	0.19	–0.08	0.337	0.05	0.24	0.02	0.839	0.14	0.17	0.07	0.414
DY2^∗^AMG fandom	–0.06	0.20	–0.02	0.774	–0.15	0.26	–0.05	0.567	0.09	0.18	0.04	0.611
Gender^∗^AMG fandom	0.34	0.16	0.16	0.039	–0.11	0.21	–0.04	0.590	–0.06	0.15	–0.03	0.677
*Step 5a*												
DY1^∗^AMG fandom^∗^gender	–0.17	0.38	–0.05	0.662	0.47	0.49	0.10	0.339	0.67	0.34	0.21	0.0498
DY2^∗^AMG fandom^∗^gender	0.11	0.42	0.02	0.800	0.08	0.54	0.01	0.883	0.11	0.38	0.03	0.767
**Japanese culture**												
*Step 3b*												
Japanese culture	–0.02	0.08	–0.01	0.815	0.05	0.10	0.03	0.638	0.10	0.07	0.08	0.179
*Step 4b*												
DY1^∗^Japanese culture	–0.29	0.19	–0.13	0.140	–0.03	0.25	–0.01	0.893	0.06	0.18	0.03	0.718
DY2^∗^Japanese culture	–0.20	0.20	–0.08	0.321	–0.16	0.26	–0.05	0.522	–0.02	0.18	–0.01	0.936
Gender^∗^Japanese culture	0.17	0.16	0.08	0.312	–0.00	0.21	–0.00	0.983	–0.10	0.15	–0.05	0.516
*Step 5b*												
DY1^∗^Japanese culture ^∗^gender	0.03	0.39	0.01	0.939	–0.07	0.50	–0.01	0.893	–0.06	0.36	–0.02	0.864
DY2^∗^Japanese culture ^∗^gender	–0.23	0.41	–0.06	0.566	0.53	0.52	0.10	0.304	0.11	0.37	0.03	0.760
**Indoor activities**												
*Step 3c*												
Indoor	–0.13	0.08	–0.09	0.114	0.30	0.10	0.17	0.003	0.20	0.07	0.16	0.007
*Step 4c*												
DY1^∗^indoor	–0.19	0.19	–0.08	0.321	0.10	0.24	0.03	0.680	0.01	0.17	0.01	0.942
DY2^∗^indoor	0.08	0.20	0.03	0.689	0.00	0.25	0.00	0.998	0.03	0.18	0.01	0.859
Gender^∗^indoor	0.15	0.16	0.07	0.361	–0.35	0.21	0.13	0.087	–0.36	0.15	–0.19	0.014
*Step 5c*												
DY1^∗^indoor^∗^gender	0.31	0.38	0.09	0.407	0.30	0.48	0.07	0.525	0.34	0.34	0.11	0.320
DY2^∗^indoor^∗^gender	1.15	0.41	0.25	0.006	–0.53	0.52	–0.09	0.315	–0.48	0.37	–0.12	0.194
**Shyness**												
*Step 3d*												
Shyness	0.12	0.08	0.09	0.121	0.06	0.10	0.03	0.581	0.08	0.07	0.07	0.243
*Step 4d*												
DY1^∗^shyness	0.17	0.19	0.08	0.361	–0.13	0.24	–0.04	0.597	–0.30	0.17	–0.16	0.072
DY2^∗^shyness	0.07	0.20	0.03	0.722	–0.19	0.26	–0.06	0.464	–0.19	0.18	–0.08	0.306
Gender^∗^shyness	0.50	0.16	0.24	0.002	–0.61	0.21	–0.22	0.004	–0.54	0.15	–0.28	<0.001
*Step 5d*												
DY1^∗^shyness^∗^gender	–0.40	0.39	–0.11	0.295	0.70	0.49	0.14	0.156	0.72	0.34	0.21	0.038
DY2^∗^shyness^∗^gender	0.01	0.41	0.00	0.985	–0.31	0.52	–0.06	0.555	–0.10	0.36	–0.03	0.775


*The experimental treatment codes and gender were entered in steps 1 and 2. In steps 3a to 5a anime, manga and games fandom (AMG fandom) was entered along with higher-order interactions. In steps 3b to 5b interest in Japanese culture (Japanese culture) was entered along with higher-order interactions. In steps 3c to 5c preference for indoor activities (indoor) was entered along with higher-order interactions. In steps 3d to 5d shyness was entered along with higher-order interactions. Experimental treatment was dummy-coded with sex robot as the comparison group. DY1 = sex robot (0) vs. nursing robot (1); DY2 = sex robot (0) vs. genetically modified organism (GMO) (1). Gender was dummy-coded (0 = male, 1 = female). All continuous predictors were z-standardized. The displayed results show the coefficients after the listed variables were entered without the influence of later steps.*

Regarding participants’ overall evaluation, the nursing robot was rated more favorably than the sex robot (*M* = 4.84, *SD* = 1.68; *M* = 3.44, *SD* = 1.68; *B* = 1.29, *SEB* = 0.25, *p* < 0.001), the sex robot did not differ from the GMO (*M* = 3.58, *SD* = 1.67; *B* = -0.01, *SEB* = 0.25, *p* = 0.98). Men gave more positive evaluations overall than women (*M* = 4.34, *SD* = 1.67; *M* = 3.41, *SD* = 1.82, *B* = -0.89, *SEB* = 0.21, *p* < 0.001). One significant interaction between technology and gender emerged: men’s more positive ratings were particularly pronounced for sex robots, as compared to GMOs, *B* = 1.03, *SEB* = 0.51, *p* = 0.044.

With respect to participants’ behavioral intentions to approach and buy the technology, the sex robot received lower scores than the nursing robot (*M* = 2.41, *SD* = 1.23; *M* = 3.33, *SD* = 1.06, *B* = 0.86, *SEB* = 0.18, *p* < 0.001), but the former did not differ from the GMO (*M* = 2.78, *SD* = 1.22; *B* = 0.30, *SEB* = 0.18, *p* = 0.10). Men reported on higher behavioral intentions overall than women (*M* = 3.04, *SD* = 1.16; *M* = 2.57, *SD* = 1.27, *B* = -0.41, *SEB* = 0.15, *p* = 0.006), however, differences between the technologies on behavioral intentions did not systematically vary with participants’ gender, *p*s > 0.18 for both interactions.

### AMG Fandom

Next we entered AMG fandom as an additional predictor to the regression equations (Step 3a). The more individuals were fans of anime, manga, and games, the less they reported on uncanny feelings regarding the technologies overall, *B* = -0.24, *SEB* = 0.08, *p* = 0.002. With respect to the two-way interactions, the kind of technology did not influence the effect of anime fandom, but gender did, *B* = 0.34, *SEB* = 0.16, *p* = 0.04: Whereas anime fandom was a significant predictor of eeriness for men (i.e., stronger fandom was associated with less uncanny feelings), *B* = -0.38, *SEB* = 0.09, *p* < 0.001, it was not a significant predictor for women, *B* = -0.04, *SEB* = 0.14, *p* = 0.79. Both three-way interactions were non-significant (*p*s > 0.66). Regarding technology evaluations, fans of anime, manga, and games, were more positive overall, *B* = 0.30, *SEB* = 0.10, *p* = 0.003. The kind of technology did not influence the effect of anime fandom on evaluations, nor did gender. Moreover, none of the other interactions was significant (all *ps* > 0.33). Our third dependent variable was behavioral intentions. Fans of anime, manga, and games, had more positive behavioral intentions overall, *B* = 0.28, *SEB* = 0.07, *p* < 0.001. The two-way interactions did not contribute to explaining behavioral intentions, but the three-way interaction between differences between nursing robots and sex robots, gender, and anime fandom did, *B* = 0.67, *SEB* = 0.34, *p* = 0.0498. For men, anime fandom was similarly associated with the behavioral intentions regarding sex robots, *r*(41) = 0.28, *p* = 0.067, and nursing robots, *r*(53) = 0.27, *p* = 0.047. For women, however, anime fandom was associated with the behavioral intentions regarding nursing robots, *r*(32) = 0.43, *p* = 0.011, but not with intentions regarding sex robots, *r*(42) = 0.02, *p* = 0.92. In sum, these findings provide mixed support for Hypotheses 1a, 1b, and 1c. AMG fandom predicted less eeriness and more purchase intentions, for men and more positive evaluations for both genders. These associations, however, were not consistently larger for sex robots as compared to both other future technologies.

### Japanese Culture

A penchant for Japanese culture was unrelated to the feelings, evaluations, and intentions regarding sex robots, nursing robots, or GMOs (Research Question 1). Neither did we find direct effects, nor two-way or three-way interactions with gender or technology (all *p*s > 0.17).

### Preference for Indoor Activities

Preference for indoor activities was unrelated to uncanny feelings overall, *B* = -0.13, *SEB* = 0.08, *p* = 0.11. The two-way interactions did not yield a significant contribution. However, a three-way interaction between the sex robot and GMO comparison, gender, and preference for indoor activities emerged, *B* = 1.15, *SEB* = 0.41, *p* = 0.006. Regarding sex robots, preference for indoor activities was neither substantially related for men *r*(41) = 0.03, *p* = 0.83, nor for women, *r*(42) = -0.17, *p* = 0.28. Regarding GMOs, higher preference for indoor activities was unrelated to uncanny feelings for men *r*(54) = -0.21, *p* = 0.11. In contrast, higher preference for indoor activities was related to more uncanny feelings with respect to GMOs for women *r*(27) = 0.40, *p* = 0.032. In sum, no support for Hypothesis 2a was found. Preference for indoor activities was positively associated with evaluations overall, *B* = 0.30, *SEB* = 0.10, *p* = 0.003, irrespective of the technology described (*p*s < 0.67), and irrespective of participants’ gender (*p* = 0.09). Likewise, no three-way interaction was significant (all *ps* > 0.32). As predicted in Hypothesis 2b, preference for indoor activities predicted evaluations, but this finding was not specific to sex robots. Preference for indoor activities was further positively associated with behavioral intentions for all three technologies (Hypothesis 2c), *B* = 0.20, *SEB* = 0.07, *p* = 0.007, with a significantly stronger relationship for men (*B* = 0.34, *SEB* = 0.09, *p* < 0.001) than for women (*B* = -0.02, *SEB* = 0.12, *p* = 0.85). Neither the interaction with the specific technology displayed nor any other interaction was significant (all *ps* > 0.13).

### Shyness

Shyness was examined as our final dimension of otakuism. Shyness was unrelated to uncanny feelings regarding the technologies overall, *B* = 0.12, *SEB* = 0.08, *p* = 0.12. With respect to the two-way interactions, the specific technology did not influence the effect of shyness, but gender determined whether or not shyness predicted uncanny feelings, *B* = 0.50, *SEB* = 0.16, *p* = 0.002: Shyness was unrelated to uncanny feelings among men, *B* = -0.07, *SEB* = 0.10, *p* = 0.47, but more shyness was associated with more uncanny feelings among women, *B* = 0.41, *SEB* = 0.13, *p* = 0.002. Both three-way interactions were non-significant (*p*s > 0.29). Thus, no support for Hypothesis 3a was established. Regarding the evaluations of the technologies, shyness was again unrelated to evaluations overall, *B* = 0.06, *SEB* = 0.10, *p* = 0.58. Among the two-way interactions, the technology interaction terms did not predict evaluations, but we found a moderation effect for gender, *B* = -0.61, *SEB* = 0.21, *p* = 0.004: For men higher shyness scores were related to more positive technology evaluations, *B* = 0.30, *SEB* = 0.12, *p* = 0.02. For women, we observed a reversed, trend-significant relationship, *B* = -0.29, *SEB* = 0.17, *p* = 0.086. Again, entering the three-way interactions did not significantly contribute to the model (*p*s > 0.16). In sum, the data are consistent with Hypothesis 3b among men, but not among women.

**FIGURE 1:**
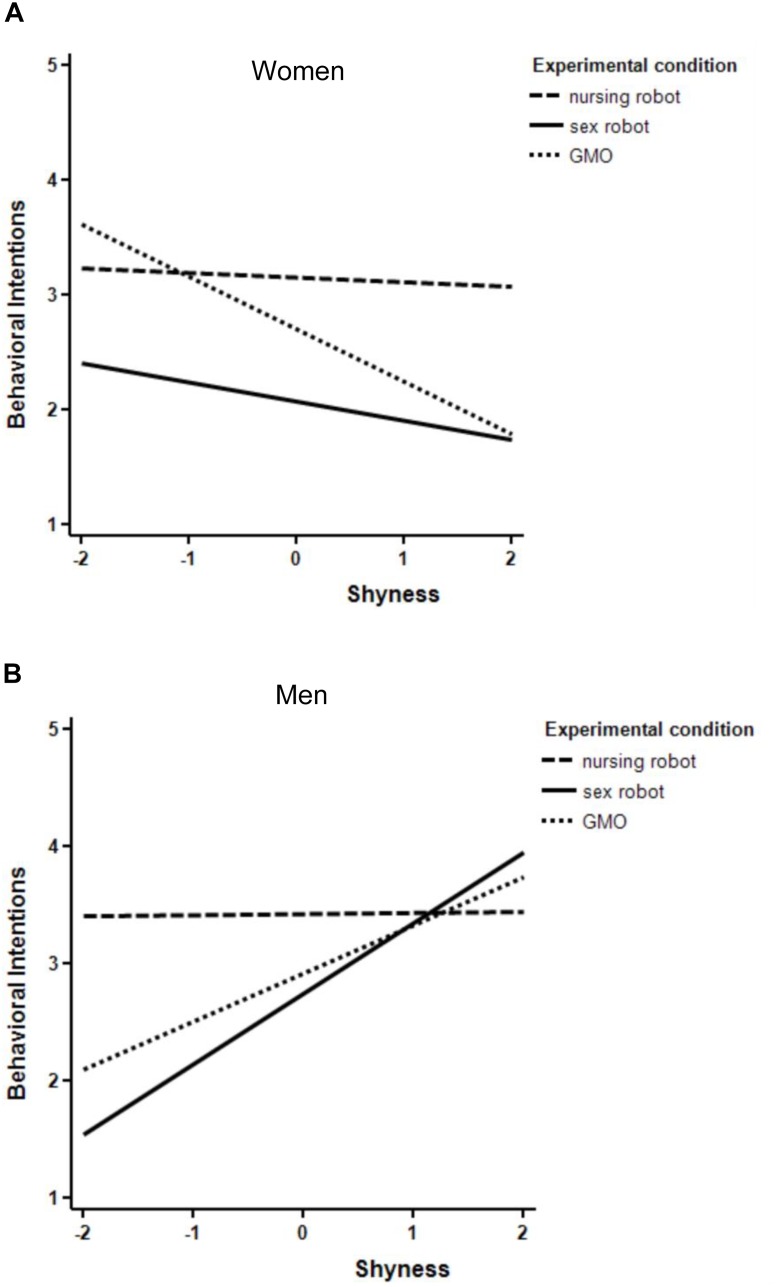
**(A)** Behavioral intentions regressed on shyness and experimental treatment, women only. **(B)** Behavioral intentions regressed on shyness and experimental treatment, men only.

Our final regression analysis addressed shyness as a predictor of the intentions to engage with the future technology. Shyness was unrelated to evaluations overall, *B* = 0.06, *SEB* = 0.10, *p* = 0.58. Whereas the two-way interactions with the technology factor did not predict behavioral intentions, gender influenced the link between shyness and behavioral intentions, *B* = -0.54, *SEB* = 0.15, *p* < 0.001. This interaction was qualified by a three-way interaction between the sex robot vs. nursing robot dummy variable, gender, and shyness, *B* = 0.72*, SEB* = 0.34, *p* = 0.038: Behavioral intentions to use a nursing robot were unrelated to shyness for women, *r*(32) = -0.03, *p* = 0.86, as well as for men, *r*(53) = 0.01, *p* = 0.93 (Figure 1A). In contrast, shyness was relevant for sex robots: Higher shyness was unrelated to behavioral intentions to use a sex robot among women *r*(42) = -0.16, *p* = 0.32, but higher shyness predicted stronger behavioral intentions to use a sex robot among men *r*(41) = 0.46, *p* = 0.002 (Figure 1B). Regarding the GMOs, higher shyness was negatively related to behavioral intentions among women *r*(27) = -0.39, *p* = 0.033, but higher shyness was positively related to behavioral intentions among men *r*(54) = 0.30, *p* = 0.025. Again, these findings provide partial support for Hypothesis 3c for men, but not for women.

## Discussion

The idea of non-human but human-like intimate companions dates back to Greek mythology. Pygmalion crafted the ivory sculpture Galatea, fell in love, and gave her life through a kiss (e.g., in Ovid’s *Metamorphoses*). Laodamia, grief-ridden after losing her husband in the Trojan wars, made a bronze statue of the late husband. She refused to remarry but was observed to embrace and kiss the statue instead (according to Hyginus’ *Fabulae*). In more recent times authors and recipients of science fiction have been fascinated by human-like machines, such as the automaton Olympia in Hoffman’s early 19th century story *The Sandman* or the synthetic men and women in the movie *Blade Runner* or the TV series *Humans* (Channel 4/AMC) and *Westworld* (HBO). The main purpose of some of these fictional humanoid creatures is to provide sexual pleasure (e.g., the male android Rick in *Humans* or the humanoid Maeve in *Westworld*). Sex robots are projected to have a substantial share in the multi-billion dollar and growing sex tech industry – although many of the current prototypes are arguably not sophisticated enough to be mass marketed (Kleeman, 2017). Besides the potential relevance to marketers, the development of sex robots gives rise to important ethical and legal questions (e.g., Richardson, 2016). Anecdotal evidence suggests that the prospect of sex robots strikes many to be eerie, initial research, however, suggests that a substantial part of individuals can imagine purchasing such a robot (Szczuka and Krämer, 2016).

The subcultural group of the otakus – for whom life size body pillows and holographic representations of their waifus are already marketed – was chosen as a fruitful starting point to examine psychological characteristics predictive of eeriness, evaluations, and behavioral intentions regarding sex robots. To disentangle perceptions and intentions toward sex robots from a general openness to social robots or to other innovations that cross category boundaries (GMOs), an experimental design was used.

Sex robots were perceived to be more eerie and more negative overall than the non-sex-related service robots (nursing robots) and our participants were less inclined to purchase the sex robot than the nursing robot. Ratings of sex robots were similar to those of the GMO. For all three innovative technologies, the three dependent variables showed that women were more reserved than men. Overall evaluations by women were particularly negative for sex robots as compared to nursing robots. This finding replicates prior results on gender differences in the acceptance of sex robots (Scheutz and Arnold, 2016) and they reflect large gender differences among the customer base of sex dolls and sex robots (Kleeman, 2017).

Our main contribution was to introduce the otakuism dimensions as predictors of sex robot appeal. The otakuism dimensions themselves were positively related among men, only to a lesser extent among women, suggesting that the otakuism network of related traits and interests is tighter for men than for women. The influence of interest in Japanese culture on the appeal of sex robots, addressed as a research question, was negligible. As expected, a penchant for anime, manga and games (AMG fandom) showed meaningful positive relationships with the appeal of sex robots, but also with the appeal of both other technologies. Thus, AMG fans are not only those who are particularly open to sex robots (as waifu body pillows suggest), they are open to all three technologies examined, highlighting that entering fictional worlds might be related to or even influence greater acceptance of threshold technologies more generally (cf. Mara and Appel, 2015; Appel et al., 2016). Likewise preference for indoor activities was related to higher evaluations and higher purchase intentions irrespective of the technology. While these findings speak to the appeal of innovative technologies among otakus in a general sense, sex robots stood out when behavioral intentions regarding sex robots and nursing robots were compared. For nursing robots AMG fandom predicted intentions for both genders, whereas AMG fandom was a stronger predictor of sex robot purchase intentions among men than among women. Shyness was a particularly relevant predictor for male purchase intentions of sex robots. Higher shyness was most strongly associated with behavioral intentions, if the robot was a sex robot and participants were men.

Note that our research was guided by a respect for otaku culture and our results provide no argument to degrade or discredit the large numbers of (self-ascribed) otakus worldwide. Our findings show that Otakus’ openness to technology is not restricted to sex robots. The relationships with AMG fandom and the preference for indoor activities hold for all three technologies. From a subcultural group of fanatic manga and anime collectors back in the 1980s, the otaku phenomenon has been spreading out widely since the turn of the millennium, mostly through highly popular anime series such as *Densha Otoko* (Galbraith et al., 2015). Since then, much of the stigma associated with being an otaku has been gradually eroding and fans of anime, manga, and video games in and outside of Japan are now defining themselves as otaku with pride (Galbraith, 2009). As an interviewee told *The Japan Times*: “‘I wouldn’t be offended if someone called me an otaku,’ says Koichi Nakayasu, ‘… because I am.”’ (McNicol, 2004). Otakuism has arrived in the heart of the popular culture, including university courses on the subject, an otaku themed pavilion at the Venice architecture biennale (The Japan Foundation, 2004), and hundreds of thousands of international tourists who visit Akihabara, Tokyo’s otaku district and now trend location, each year. However, if there is a growing number of young people who seem to prefer fictional worlds over day-to-day reality, virtual friends over physical ones, and, as our research indicates, possibly also sex robots over human partners, future research will also have to deal with the origins and mechanisms behind these trends.

### Limitations and Outlook

Despite the contribution of our work, limitations need to be noted. First, we did not examine real human–robot interactions but potential future users’ responses to robotic innovations that were presented in text. This was a feasible way to approach the topic empirically, as the first professionally made sex robots were not yet available to the public when this manuscript was written (the test-launch of *Harmony* by Abyss Creation has ostensibly started in the end of 2018; the sex robot Roxxxy, which has attracted a lot of media fuzz, has apparently never been distributed, cf. Kleeman, 2017). We believe that our results hold for real human-robot interactions in the field, but future studies are encouraged to test this prediction empirically.

We did not ask participants whether or not they would self-ascribe being an otaku. Theoretically, a participant may have scored highly on all four dimensions we investigated (AMG fandom, preference for indoor activities, shyness, and interest in Japanese culture), but would not have perceived him- or herself as an otaku. Part of our United States participants may even have never heard of this term. Otakuism had been the starting point of our investigation, but the core of theoretical and empirical work was on the four dimensions that were empirically investigated. We believe that this approach is defensible, but it needs to be acknowledged.

The otakuism complex we investigated is likely not the only set of individual difference variables that could predict the acceptance and appeal of sex robots. Attitudes toward technology or robots and the familiarity with mass-marketed virtual assistants like *Alexa* or *Siri* could be a fruitful field of inquiry (cf. Szczuka and Krämer, 2017). Regarding personality, the *dark tetrad* (Buckels et al., 2013; Paulhus, 2014), for example, could be an additional contributor. The *dark tetrad* of personalities is a set of undesirable, relatively stable, subclinical characteristics of human perception and behavior. It consists of the traits narcissism, Machiavellianism, psychopathy, and sadism. The common feature of these traits is a lack of empathy toward others. We assume that both narcissism and sadism could predict the appeal of sex robots. Narcissism is characterized by an inflated sense of the self and grandiose self-promotion. Narcissists continually crave attention; they perceive themselves as gifted and remarkable and they need others to demonstrate their supposedly superior qualities and achievements (e.g., Wallace and Baumeister, 2002). Narcissism is associated with high, but vulnerable self-esteem, which can easily be threatened by other humans (Bushman and Baumeister, 1998). Sex robots, whose artificial intelligence is programmed to flatter the user, could consistently reinforce the embellished self-concept of narcissists, which narcissists – given their distorted self-view – would arguably find particularly believable and attractive (John and Robins, 1994). Moreover sadism could predict the appeal of sex robots. Sadism, or the enjoyment of cruelty, could motivate behavioral manifestations of sexual desires that would severely harm a human partner (Kirsch and Becker, 2007). Sex robots, however, could be perceived as welcome and relatively risk-free counterparts by sadists. Moreover, sex robots could help fulfill sadistic tendencies outside the immediate sexual field.

Finally, we want to emphasize that there is a bunch of potential social, legal and moral implications of sex robots that will need to be discussed in the years ahead and that require the involvement of various stakeholders and scholarly disciplines. Concerns that have been raised about sex robots before, especially about highly lifelike and technologically sophisticated ones, address privacy and security risks, a potential growth of social isolation, emotional manipulation of lonely and vulnerable persons, objectification of the human body, the reinforcement of traditional gender-related stereotypes and the potential promotion of sexual violence and pedophilia (Sullins, 2012; Richardson, 2016; Sharkey et al., 2017). We do not go into detail about these issues, because they are beyond the scope of this research article. Nevertheless, we consider them very important and encourage everybody to think about the implications of sex robots for future societies.

## Conclusion

The more people are into manga and games and the more they prefer to stay inside – core aspects of being an otaku – the higher the appeal of sex robots. These relationships were not more pronounced for sex robots than for two other technological innovations (nursing robots, GMOs), however. Shyness was a particularly relevant predictor for men’s purchase intentions of sex robots (as compared to nursing robots). These findings on sex robot appeal are relevant to researchers as well as the general public, as the advent of sex robots is projected to revolutionize interpersonal relationships in the not too far future.

## Ethics Statement

The experiment was approved by the IRB board of the Human-Computer-Media Institute, University of Würzburg, Germany.

## Author Contributions

MA, CM, and MM contributed to the conception and design of the study. MA performed the statistical analysis and wrote the first draft of the manuscript. All authors contributed to manuscript revision, read and approved the submitted version.

## Conflict of Interest Statement

The authors declare that the research was conducted in the absence of any commercial or financial relationships that could be construed as a potential conflict of interest.
