# Supplementary material for: Otakuism and the Appeal of Sex Robots
Source: Front Psychol. 2019 Mar 29;10:569. doi: 10.3389/fpsyg.2019.00569 (PMC6449875; doi:10.3389/fpsyg.2019.00569)
Supplement: Supplementary file 1 [file Data_Sheet_1.docx]

Appendix

Participants were randomly assigned to read one of the following three descriptions:

1)

We are interested in your thoughts on a new generation of robots.

Ellix is a nursing robot with arms and hands. Ellix was designed to look like and feel like a human being. There are two variants of this robot, one is male and one is female. Ellix assists elderly people who are in need of support. One of its main tasks is to feed people.

2)

We are interested in your thoughts on a new generation of robots.

Ellix is a companion robot with arms and hands. Ellix was designed to look like and feel like a human being. There are two variants of this robot, one is male and one is female. Ellix is made for leisure activities and entertainment. One of its main tasks is to provide sexual pleasure.

3)

We are interested in your thoughts on a new generation of foods.

Ellix is the name for a genetically modified organism. Ellix was designed to combine features of plants and animals. Ellix can be grown like a plant, but the genes include those found in animals. It has high nutritional value, a taste that many consumers prefer over the taste of vegetables, and it facilitates the work of farmers.
