# Supplementary material for: Otakuism and the Appeal of Sex Robots
Source: Front Psychol. 2019 Mar 29;10:569. doi: 10.3389/fpsyg.2019.00569 (PMC6449875; doi:10.3389/fpsyg.2019.00569)
Supplement: Supplementary file 2 [file Data_Sheet_2.docx]

Table S1.

*Supplemental analysis with otakuism dimensions as predictors entered together in Step 3*

|  |  | **Eeriness** | | | |  | **Evaluation** | | | |  | **Purchase Intentions** | | | |
| --- | --- | --- | --- | --- | --- | --- | --- | --- | --- | --- | --- | --- | --- | --- | --- |
|  |  | B | SE_B_ | β | *p* |  | B | SE_B_ | β | *p* |  | B | SE_B_ | β | *p* |
| **Technology / Gender** |  |  |  |  |  |  |  |  |  |  |  |  |  |  |  |
| *Step 1* |  |  |  |  |  |  |  |  |  |  |  |  |  |  |  |
| Gender |  | 0.54 | 0.16 | .19 | .001 |  | -0.89 | 0.21 | -.24 | <.001 |  | -0.41 | 0.15 | -.16 | .006 |
| DY1 Sex robot vs. nursing robot |  | -0.91 | 0.19 | -.32 | <.001 |  | 1.29 | 0.25 | .34 | <.001 |  | 0.86 | 0.18 | .33 | <.001 |
| DY2 Sex robot vs. GMO |  | -0.10 | 0.20 | -.04 | .599 |  | -0.01 | 0.25 | -.00 | .981 |  | 0.30 | 0.18 | .11 | .098 |
| *Step 2* |  |  |  |  |  |  |  |  |  |  |  |  |  |  |  |
| DY1*Gender |  | -0.42 | 0.39 | -.10 | .288 |  | 0.46 | 0.50 | .09 | .352 |  | 0.45 | 0.35 | .12 | .209 |
| DY2*Gender |  | -0.32 | 0.40 | -.08 | .419 |  | 1.03 | 0.51 | .18 | .044 |  | 0.49 | 0.36 | .13 | .176 |
|  |  |  |  |  |  |  |  |  |  |  |  |  |  |  |  |
| **Otakuism** |  |  |  |  |  |  |  |  |  |  |  |  |  |  |  |
| *Alternative Step 3* |  |  |  |  |  |  |  |  |  |  |  |  |  |  |  |
| AMG fandom |  | -0.34 | 0.09 | -.25 | <.001 |  | 0.32 | 0.12 | .18 | .008 |  | 0.27 | 0.09 | .22 | .002 |
| Japanese culture |  | 0.15 | 0.09 | .11 | .093 |  | -0.15 | 0.12 | -.08 | .209 |  | -0.06 | 0.08 | -.05 | .468 |
| Indoor activities |  | -0.14 | 0.09 | -.10 | .109 |  | 0.27 | 0.11 | .15 | .016 |  | 0.13 | 0.08 | .11 | .094 |
| Shyness |  | 0.25 | 0.08 | .18 | .004 |  | -0.11 | 0.11 | -.06 | .307 |  | -0.03 | 0.08 | -.02 | .729 |
